# Comparison of Preoperative Assessment of Patient's Metabolic Equivalents (METs) Estimated from History versus Measured by Exercise Cardiac Stress Testing

**DOI:** 10.1155/2018/5912726

**Published:** 2018-09-03

**Authors:** Adam S. Weinstein, Martin I. Sigurdsson, Angela M. Bader

**Affiliations:** ^1^Department of Anesthesiology, Perioperative and Pain Medicine Brigham and Women's Hospital, 75 Francis Street, CWN-L1, Boston, MA 02115, USA; ^2^Department of Anesthesiology, Duke University, 2301 Erwin Road, Durham, NC 027701, USA

## Abstract

**Background:**

Preoperative anesthetic evaluations of patients before surgery traditionally involves assessment of a patient's functional capacity to estimate perioperative risk of cardiovascular complications and need for further workup. This is typically done by inquiring about the patient's physical activity, with the goal of providing an estimate of the metabolic equivalents (METs) that the patient can perform without signs of myocardial ischemia or cardiac failure. We sought to compare estimates of patients' METs between preoperative assessment by medical history with quantified assessment of METs via the exercise cardiac stress test.

**Methods:**

A single-center retrospective chart review from 12/1/2005 to 5/31/2015 was performed on 492 patients who had preoperative evaluations with a cardiac stress test ordered by a perioperative anesthesiologist. Of those, a total of 170 charts were identified as having a preoperative evaluation note and an exercise cardiac stress test. The METs of the patient estimated by history and the METs quantified by the exercise cardiac stress test were compared using a Bland–Altman plot and Cohen's kappa.

**Results:**

Exercise cardiac stress test quantified METs were on average 3.3 METS higher than the METs estimated by the preoperative evaluation history. Only 9% of patients had lower METs quantified by the cardiac stress test than by history.

**Conclusions:**

The METs of a patient estimated by preoperative history often underestimates the METs measured by exercise stress testing. This demonstrates that the preoperative assessments of patients' METs are often conservative which errs on the side of patient safety as it lowers the threshold for deciding to order further cardiac stress testing for screening for ischemia or cardiac failure.

## 1. Background

One metabolic equivalent (MET) is defined as the basal oxygen consumption of a 40-year-old 70 kg man [1]. The maximum amount of metabolic work that an individual can perform can be described in MET units and this corresponds to overall cardiovascular fitness. These measurements have been utilized in preoperative assessments of patients where a patient's functional capacity is described in METs and for risk stratification for perioperative complications for patients undergoing noncardiac surgical procedures. Traditionally, a patient's maximum METs are quantified by querying them for a description of their physical activities and using activity scales, which correlates a physical activity with a quantified number of METs [2–4]. This assessment provides an estimate of a patient's METs that is assumed to correspond to a formal quantitative measurement of METs performed during a cardiac exercise stress test.

It is well known that patients with low METs are at increased risk for perioperative morbidity and mortality [1, 2, 5, 6]. An accurate preoperative assessment of a patient's METs is important. If a patient cannot perform four METs, this could prompt further cardiac workup by the Stepwise Approach to Perioperative Cardiac Assessment Treatment algorithm from the ACC/AHA Guidelines on Perioperative Cardiovascular Evaluation and Management of Patients Undergoing Noncardiac Surgery, because these patients have increased postoperative complications [1]. Previous studies have focused on quantifying physical activities with quantified METs and correlating the METs to outcomes. The focus of this study was the accuracy of the patient's METs obtained by history in the anesthesia preoperative evaluation.

Therefore, we performed a single-center retrospective study over a ten-year period to examine the accuracy of anesthesiologists' assessments of patient's METs in the anesthesia preoperative evaluation by comparing the preoperative metabolic equivalents (METs) estimated from history to the formally quantified METs during exercise cardiac stress testing.

## 2. Methods

The Brigham and Women's Hospital (BWH) Institutional Review Board (IRB) granted approval for the chart review and waived individual consent. BWH uses the computer software program Precipio to order cardiac stress tests. Using this software, we identified patients that underwent cardiac stress tests ordered by an anesthesiologist at the BWH preoperative evaluation clinic from 12/1/2005 to 5/31/2015. Clinical data were extracted from the medical charts in a de-identified manner and stored in an encrypted and password protected excel spreadsheet that was created specifically for this study.

Medical charts with patients undergoing cardiac stress tests without an available preoperative anesthesia evaluation note (*N*=96) or an incomplete anesthesia evaluation note (*N*=28) were excluded. The types of cardiac stress tests, exercise or nonexercise, were assessed. For those patients that had exercise cardiac stress tests (*N*=170), we noted the METs of the patient estimated by history and the METs quantified by the exercise cardiac stress test following the visit. Exercise cardiac stress tests at BWH use the Bruce Protocol for quantification of METs. During preoperative assessments at the BWH preoperative evaluation clinic, the METs of a patient are estimated by history using the BWH preoperative evaluation clinic METs Table (Table 1), which gives examples of physical activities and their MET equivalents based on known standards.

The two METs assessments were compared using a Bland–Altman plot and by calculating a Spearman's correlation. Furthermore, distribution of METs values into categories of poor (less than 4 METs), moderate (4–6 METs), good (7–10 METs), and excellent (10 or higher METs) was compared between the two assessments using Cohen's kappa. Calculations were done in R (version 3.0, The R foundation, Austria), and a *p* value < 0.05 was considered significant. Comparison was repeated after excluding all patients that had an estimated MET by history of four (*N*=53) to eliminate error from inadequate full assessment of a patients full MET potential since 4 METs is a decision point in the ACC/AHA Guidelines.

## 3. Results

**Figure 1:**
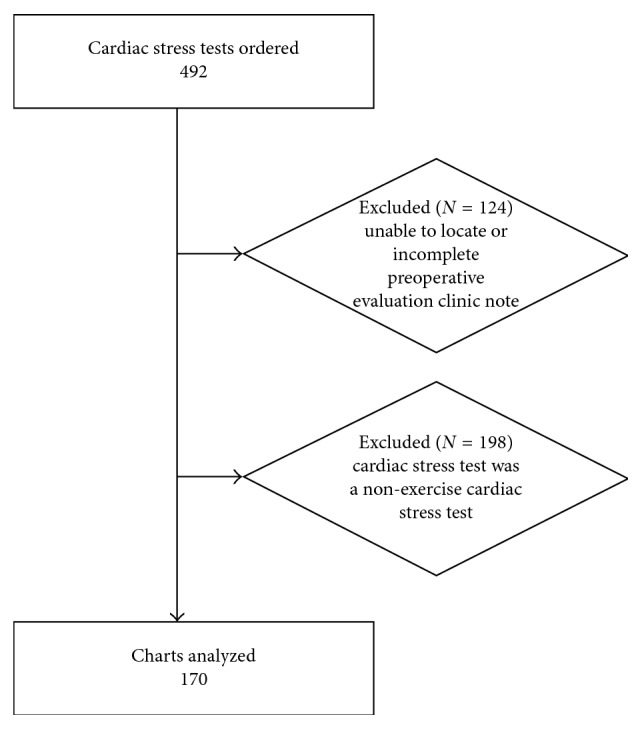
Flow diagram of the charts analyzed and excluded.

A total of 492 cardiac stress tests (one per patient) ordered by an anesthesiologist at the BWH preoperative evaluation clinic from 12/1/2005 to 5/31/2015 were identified. For 124 of the cardiac stress tests ordered, the accompanying preoperative evaluation clinic visit notes were unobtainable or incomplete and were not analyzed for indication. At the study's institution, the transition in 2015 from paper-based medical records to electronic medical records resulted in a loss of some original paper-based notes and some preoperative evaluation clinic visit notes not obtainable either due to them not ever being scanned in, incompletely scanned in, or misclassified as a different note type in the electronic medical record. The remaining 368 cardiac stress tests were analyzed, and of those, 198 were identified as nonexercise cardiac stress tests as the cardiac stress test modality, leaving 170 charts for final analysis (Figure 1).

The patient demographics are shown in Table 2.

**Figure 2:**
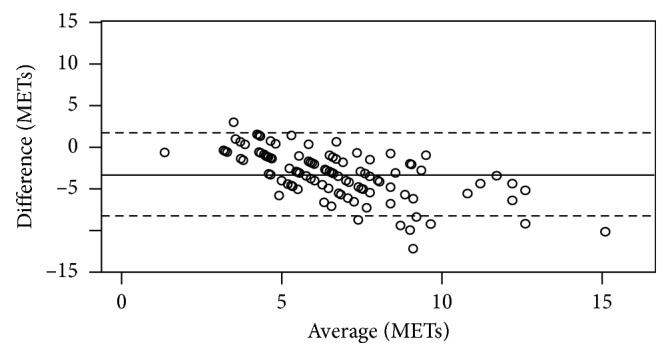
Bland–Altman plot of METs estimated from history versus measured by exercise cardiac stress testing.

Mean and median estimated METs by history were 4.9 ± 1.6 and 5 respectively, and mean and median measured METs by the cardiac stress test were 8.3 ± 3.0 and 7.8, respectively. Although there was a modest correlation between the two measurements (Spearman's rho 0.37, *p* < 0.001), the agreement between the two methods was generally poor (Figure 2).

The exercise cardiac stress test measured METs was on average 3.3 METs higher than the METs estimated from the preoperative evaluation clinic history. Only 16 (9%) patients had lower METs quantified by exercise cardiac stress test than estimated by history. The agreement between the two methods compared between patient groups of poor (less than 4 METs), moderate (4–6 METs), good (7–10 METs), and excellent (10 or higher METs) functional capacity was very poor (Cohen's kappa 0.02, *p*=0.446). Of the 170 patients, the categorization of the patients' METs by history and exercise cardiac stress test into poor, moderate, good, or excellent agreed for 38 (22%) of the patients. 124 (73%) patients had a higher functional capacity grouping by measured METs by exercise cardiac stress test than METs quantified by history. Only 8 (5%) patients had a lower functional capacity grouping by measured METs by exercise cardiac stress test than METs estimated by history. In addition, only 7 (4%) patients had measured METs by the exercise cardiac stress test of less than 4 and METs estimated by history of greater than 4. Repeating the analysis but excluding patients with a preoperative assessment visit note that estimated 4 METs by history revealed similar results (data not shown).

## 4. Discussion

We performed a single-center retrospective study of 170 charts over a 10-year period comparing the preoperative METs estimated from history to METs quantified during exercise cardiac stress testing. We found that, on average, the METS measured by the preoperative exercise cardiac stress test was 3.3 METs higher than the METs estimated by preoperative history at the preoperative evaluation clinic visit. The Bland–Altman plot also demonstrates that generally the METs estimated by history are less than the METs measured by the exercise cardiac stress test with increasing dispersion the higher the MET capacity of the patient.

There are limitations to this study. The assessment of METs was only semi-structured, meaning the clinician interviewer was provided a list of example activities and their associated METs, but the recorded assessment was at the discretion of the clinician. Since this was a retrospective study, we were unable to control for variables such as the ability of the preoperative physician to adequately ascertain the patient's METs from history and the patient's knowledge of his or her exercise capacity. Furthermore, we could not adjust for the practice of some preoperative clinicians to document 4 METs even if the patient is able to achieve higher METs. To help control for the latter limitation, we excluded patients whose METs obtained by preoperative history at the preoperative evaluation visit were documented as 4. Using this subset, the average METS measured by the preoperative exercise cardiac stress was still 3.3 METs higher than the METs estimated by preoperative history at the preoperative evaluation clinic visit.

Another limitation is that the indication for cardiac stress testing is not random. There are many reasons to order a cardiac stress test preoperatively, which is a vast topic of its own and outside the scope of this article. Looking at the table for the indications (Table 2) for which clinicians in this study ordered cardiac stress tests (METs < 4, ECG findings, chest pain, and other), it is evident that the indications are cardiovascular related. In addition, the inability to perform 4 METs or if it unknown whether 4 METs can be performed is a strong indication for ordering a cardiac stress test if other criteria are met. An alternate perspective on this information is that a patient who's METs is greater than four, or exercises and knows their activity level well, is less likely to get a preoperative cardiac stress test. A reasonable assumption can be made that patients who had cardiac stress tests ordered compared with those who did not are more likely to have cardiovascular pathologies and be less fit.

There are published recommendations and appropriate use criteria for specific types of cardiac stress tests [7–10]; however, there is no consensus or recommendation about which the modality of cardiac stress test to choose preoperatively. This decision is up to the discretion of the ordering clinician. There are multiple reasons why exercise versus nonexercise cardiac stress testing may have been chosen by the ordering clinician which were uncontrollable since the study is retrospective. This may have led to a selection bias in the study since patient-specific factors likely influenced the ordering clinician's choice between an exercise and nonexercise cardiac stress test creating differences in the two patient populations. These differences may derive from the following: First, a proportion of preoperative cardiac stress tests are a result of the Stepwise Approach to Perioperative Cardiac Assessment Treatment algorithm from the ACC/AHA Guidelines on Perioperative Cardiovascular Evaluation and Management of Patients Undergoing Noncardiac Surgery [1]. Although these guidelines have evolved from 2002 to 2014, the ability of a patient to perform 4 METs has remained an important decision point in the stepwise algorithm for cardiac assessment. The inability to perform 4 METs or if it unknown whether 4 METs can be performed is a strong indication for ordering a cardiac stress test if other criteria are met. In these patients, the most recent ACC/AHA Guidelines (2014) recommends specifically pharmacologic (nonexercise) stress testing, which pertains to only a small portion of this study (since the ACC/AHA Guidelines were updated in December 2014 and this retrospective study ended in May 2015). Prior to the 2014 Guidelines, the decision of exercise versus the nonexercise cardiac stress test was up to the ordering clinician. Thus, from December 2014 to May 2015 there is a bias for more pharmacologic stress tests in patients whose preoperative estimate of METS is 4 or less or unknown. Second, for clinical scenarios not specifically addressed by the Stepwise Approach to Perioperative Cardiac Assessment Treatment algorithm from the updated 2014 ACC/AHA Guidelines, the decision on exercise versus nonexercise cardiac stress test is up to the clinician. Examples of this include patients with a history of atypical chest pain preoperatively or concerning ECG findings on a preoperative ECG. From these considerations there may be selection bias for a nonexercise versus exercise cardiac stress test based on the patient's history which is frequently dictated by whether the patient has physical or orthopedic commodities that limit them from exercising. A preoperative assessment of low METs due to a cardiovascular limitation rather than an orthopedic limitation does not necessitate a nonexercise cardiac stress test. Typically, all that is required for the patient to achieve a satisfactory workload, which can be defined by many variables, is to achieve 85% of the age predicted maximum heart rate [11]. In this study there clearly is a difference in the preoperative estimate of METs between the exercise and nonexercise stress test patients. The average METs estimated by history of patients who had an exercise cardiac stress test compared with those who had a nonexercise cardiac stress test were 4.9 and 3.6, respectively. In addition, the percentage of patients whose METs estimated by history were less than 4 who had an exercise cardiac stress test compared with those who had a nonexercise cardiac stress test were 15% and 49%, respectively. This difference in patients who had an exercise versus nonexercise cardiac stress test could be a limitation of this study. It can be inferred that clinicians in this study favored nonexercise cardiac stress tests in patients with a low preoperative estimate of METs.

Furthermore, this study was a single-center study. While the BWH preoperative evaluation clinic is a high-volume clinic that sees over 25,000 patients a year and has been in place for a relatively long time, this study examines only a single institution's clinicians. Therefore, our findings cannot be readily generalized to all preoperative anesthesiologists.

Last, the evaluation of METs of a patient using known standards of activities has some limitations. While published standards of METs of specific activities have been validated, the most well known being the Duke Activity Scale Index, the estimation of METs by history relies on the ability of the clinician to adequately assess the patient [3, 12]. The ability of a patient to know their activity levels and the clinician to accurately obtain that information is the challenge. Less likely is a recalibration of the validated instrument tool needed, but rather a reassessment of a clinician's ability to get an accurate functional assessment from a patient's history of activities.

## 5. Conclusions

In summary, the METs of a patient estimated by preoperative history is conservatively assessed and often underestimates the METs measured by exercise cardiac stress testing. A conservative estimate of a patients METs lowers the threshold for ordering preoperative cardiac stress tests to assess for underlying ischemia, which is a known risk for perioperative complications.

## Figures and Tables

**Table 1:** Activities and their MET equivalents.

METs	Activity
1	Eating, getting dressed, working at a desk
2	Showering, walking down eight steps
3	Walking on a flat surface for one or two blocks
4	Raking leaves, weeding or pushing a power mower, walking up two flights of stairs
5	Walking four miles per hour, social dancing, washing a car
6	Nine holes of golf carrying clubs, heavy carpentry using a push mower
7	Digging, spading soil, singles tennis, carrying 60 pounds
8	Moving heavy furniture, jogging slowly, rapidly climbing stairs, carrying 20 pounds upstairs
9	Bicycling at a moderate pace, sawing wood, slow jumping rope
10	Brisk swimming, bicycling uphill, walking briskly, uphill jogs 6 mph
11	Cross-country skiing, full-court basketball
12	Running continuously at 8 mph

**Table 2:** Patient demographics.

Patient Demographics	Mean (SD)/*N* (%)
Age	61 ± 11
Male gender	75 (44)

*Type of surgery or procedure*	*N(%)*
General	51 (30)
Gastroenterology	1 (1)
Gynecology	30 (18)
Neurosurgery	8 (5)
Orthopedic	14 (8)
Otolaryngology	15 (9)
Thoracic	22 (13)
Urology	26 (15)
Vascular	3 (2)

*Indication for stress test*	*N(%)*
ECG findings warranted stress test	99 (58)
Chest pain	37 (22)
METs less than 4	15 (9)
Stress test recommended by other medical service	11 (6)
Other	8 (5)

## Data Availability

The datasets used and/or analyzed during the current study are not publicly available due to their current use on other research projects but are available from the corresponding author on reasonable request.
